# Computational Study on the Conformation and Vibration Frequencies of β-Sheet of ɛ-Polylysine in Vacuum

**DOI:** 10.3390/ijms10083358

**Published:** 2009-07-29

**Authors:** Shiru Jia, Zhiwen Mo, Yujie Dai, Xiuli Zhang, Hongjiang Yang, Yuhua Qi

**Affiliations:** 1 Key Laboratory of Industrial Microbiology, Ministry of Education, College of Biotechnology, Tianjin University of Science and Technology, Tianjin 300457, China; E-Mails: yjdai@126.com (Y.D.); hongjiangyang@tust.edu.cn (H.Y.); 2 Department of Biochemistry, University of Missouri-Columbia, Columbia, MO 65211, USA; E-Mail: zxl1966@hotmail.com (X.Z.); 3 Institute of Theoretical Chemistry, School of Chemistry and Chemical Engineering, Shandong University, Jinan 250100, China; E-Mail: fdc@sdu.edu.cn (Y.Q.); 4 School of Chemistry and Chemical Engineering, University of Jinan, Jinan 250022, China

**Keywords:** cyclohepta bifurcated hydrogen bond, ɛ-polylysine, peptide, ONIOM

## Abstract

Two oligomers, each containing 3 l-lysine residues, were used as model molecules for the simulation of the β-sheet conformation of ɛ-polylysine (ɛ-PLL) chains. Their C terminals were capped with ethylamine and N terminals were capped with α-l-aminobutanoic acid, respectively. The calculations were carried out with the hybrid two-level ONOIM (B3LYP/6-31G:PM3) computational chemistry method. The optimized conformation was obtained and IR frequencies were compared with experimental data. The result indicated that the two chains were winded around each other to form a distinct cyclohepta structure through bifurcated hydrogen bonds. The groups of amide and α-amidocyanogen coming from one chain and the carbonyl group from the other chain were involved in the cyclohepta structure. The bond angle of the bifurcated hydrogen bonds was 66.6°. The frequency analysis at ONIOM [B3LYP/6-31G (d):PM3] level showed the IR absorbances of the main groups, such as the amide and amidocyanogen groups, were in accordance with the experimental data.

## 1. Introduction

l-Lysine is a kind of basic amino acid containing one carboxyl and two amino groups. Its carboxyl can combine with α or ɛ amino group of another l-lysine to form a cationic homopolmer, poly-l-lysine (PLL). This kind of cationic homopolymer can be used as emulsifying or delivery agents [1,2], anti-obese regents [3], hydrogels [4], biodegradable materials [5], food preservatives [6] and so on. Because α-PLL can take on different conformations such as α-helix, β-sheet, β-turn and random coil, there are many publications using it as a model to examine the structure of proteins [7–10]. The investigating methods, consisting of Fourier Transfer Infrared Spectra (FT-IR), Raman Spectra, Circular Dichroism (CD) and Nuclear Magnetic Resonance (NMR), etc, are usually used.

Compared with α-PLL, there are fewer studies on ɛ-PLL. ɛ-PLL was first discovered in the culture filtrate of an actinomycete, *Streptomyces albulus* 346, which was isolated from soil [11]. ɛ-PL can inhibit the growth of a wide range of microorganisms, including Gram-negative and Gram-positive bacteria, yeasts and molds [12–15]. Because of its notable biological activity, stability and non toxicity to humans, it is now industrially produced in Japan as a food preservative [16]. The proposed mechanism for the antimicrobial activity of ɛ-PLL may be attributed to its electrostatic adsorption onto the cell surface of microorganisms leading to stripping of the outer membrane and abnormal distribution of cytoplasm. A chain length of at least 10 l-lysine monomers was found to be optimum for the antimicrobial activity of ɛ-PLL and chemical modification of the amino groups of ɛ-PLL lowered its antibacterial activity [12]. This phenomenon shows that the structure of the polylysine has an important effect on its antimicrobial activity. It was also reported that the antibacterial activity of some cationic peptide depends on its conformation [17]. Thus, the study on the conformation, inter- and intra- molecular interactions of ɛ-PLL can expand considerably the pool of peptide foldamers, also it may provide a new comprehension to the antimicrobial mechanism of peptides.

The structures of ɛ-polylysine have been investigated with different methods. IR and CD spectroscopic studies showed that ɛ-lysine oligomers form a β-sheet structure in aqueous solution, the content of which is dependent on the chain length and pH [18]. In solid state, the parallel β-sheet conformation of ɛ-PLL similar to that of γ-type nylon-6 was demonstrated with FT-IR, Raman, and solid-state ^13^C-NMR spectra analyses [19]. However, because there is no crystal structure data available, little detailed information was obtained on the geometrical structure, inter- and intra- molecular interactions of ɛ-PLLs.

Recently, with the development of the computer hardware and software, the computation studies on molecular structures and properties are increasing rapidly. It has been an important method for the investigation of the molecular structure, IR, Raman and NMR spectra, intra-molecular and inter-molecular interactions [20–22]. However, because there are large numbers of atoms in biomacro-molecules, the high accuracy computations are very time-consuming, for example, the ab initio quantum chemical computation methods, in particular those that cover most of the electron correlation, tend to give the accurate energetics. Unfortunately, *ab initio* calculations are expensive in tradition. The computational cost of spin-restricted Hartree-Fock theory, among the least expensive of methods, scales with three power of the total atom number in the system [23]. The enormous size of protein systems then renders ab initio calculations intractable.

Generally, reduced scaling correlated methods have been developed in order to lower the expenses of the high accuracy quantum chemistry method. However, reduced scaling correlated methods remain in limited circulation and have some problems associated with geometry optimizations of hydrogen-bonded complexes [24]. In order to avoid large calculations, computational chemists study large systems in the following three ways: (1) using the cheap semi-empirical or molecular mechanical methods to study large systems [25,26]; (2) using the periodic boundary conditions to mimic the large periodic systems [27,28]; (3) using the more accurate quantum chemistry methods to study small model molecules designed to mimic the behavior of the large real system [29–31]. The emerging hybrid quantum chemical/molecular mechanical method, ONIOM, incorporating the advantages of both approaches has been set up recently. It has been used extensively for the calculation of macromolecules in biosystems [32–35]. ONION divides the system into up to three segments which can deal with complicated calculations at different levels. The essential part of the system can be treated at high level, while the less critical parts of the system might be calculated at the medium or low level. For peptide systems, usually the non-polar groups such as methyl, methylenes are at high level, and the polar groups, for example, the carbonyl, hydroxyl, amidocyanogen, are at the low level [36,37].

In this study, the geometry structures, the intra- and inter- molecular interactions of ɛ-PLLs in vacuum were studied with ONIOM computational chemistry methods and some results were compared with experimental data.

## 2. Results and Discussion

**Figure 1.**
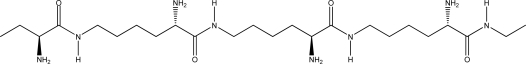
Sketch of the model compound A.

Because there are large numbers of amide bonds and α-amino groups in the ɛ-PLL molecule, the prediction of its secondary or tertiary structure is very complicated. In order to simplify the computation process, two oligomers, each having three l-lysine residues, were chosen as model molecules to simulate the interaction of ɛ-PLL chains with high degree of polymerization. The C terminal of the oligomer was capped with ethylamine, and its N terminal was capped with α-l-aminobutanoic acid (compound A, Figure 1).

### 2.1. Geometry and peptide combination of the ɛ-PLL double chains

**Figure 2.**
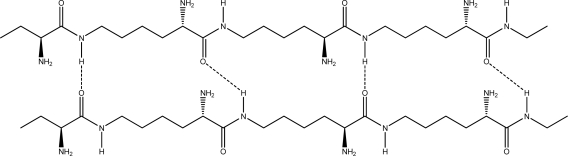

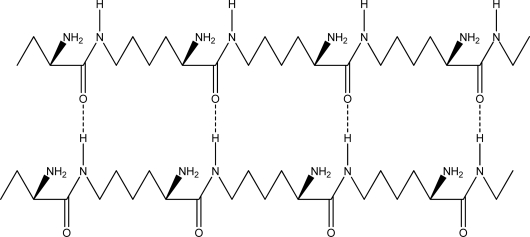
Parallel β-sheet form of ɛ-PLL double chains. (a). Proton donors and acceptors are arranged in the chains alternately. (b). Proton donors and acceptors are arranged in the separate chains.

**Figure 3.**
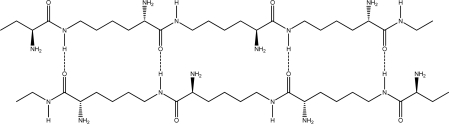

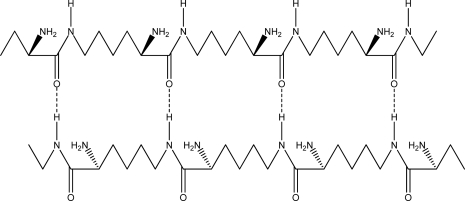
Antiparallel β-sheet form of ɛ-PLL double chains. (a). Proton donors and acceptors are arranged in the chains alternately. (b). Proton donors and acceptors are arranged in the separate chains.

Because of the large atom amounts and the flexibility of 1,6-amide skeleton in ɛ-PLLs, many different optimum conformations may be formed in the natural state. Typical conformations for the two ɛ-PLL molecule chains were simulated mainly through combinations of hydrogen bonds. Three main arrangements can be obtained according to the different arrangements of the C and N terminals:

1. Random coiled form - there is no order of the hydrogen bond combinations between the two ɛ-PLL molecules and the two chains are random coiled.
2. Parallel β-sheet form - the two molecular chains are arranged in parallel. The C terminals of the double chains are at one end, while the N terminals are at the opposite [Figures 2 (a) and (b)].
3. Anti-parallel β-sheet form - the C terminal of one chain combines with the N terminal of the other one, and the two mono-chains are arranged in the opposite direction [Figures 3 (a) and (b)].

**Figure 4.**
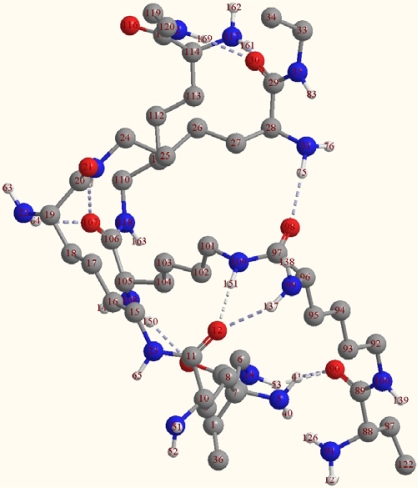
Optimized conformation of the double chains of compound A obtained from ONIOM (B3LYP/6-31G:PM3).

Many inter-molecular hydrogen bonds are formed between two ɛ-PLL chains. These hydrogen bonds can exist between the carboxyl, carbonyl, amide, and α-amino groups. Because the terminal functional groups, such as carboxyl and ɛ-amino groups, constitute only a small portion of the total in ɛ-PLLs, we mainly focused on the inter-molecular hydrogen bonds existing between C=O and H-N in the intermediate part of the chains and neglected the interaction of the terminal groups of the ɛ-PLL chains. For each circumstance of (2) or (3), the conformation of the double-chain may have two subforms according to the different arrangement of inter-molecular hydrogen bonds: (I) Proton donors (H-N bonds in amide or amino group) and acceptors (O=C groups) are arranged in the two chains alternately [Figure 2(a) and Figure 3(a)]; (II) Proton donors (H-N bonds in amide or amino group) and acceptors are located at separate chains [Figure 2(b) and Figure 3(b)]. In fact, for the two long chains of ɛ-PLLs with high molecular weight, the proton donors and acceptors may be arranged randomly as a whole, but there may be some sections where the proton donors and acceptors are arranged regularly. From the two dimensional sketches of the double chains, we might conclude that dimer 3a [Figure 3(a)] is the most stable conformation, while dimers 2b and 3b [Figure 2(b) and 3(b) are the least suitable arrangements in space matching. However, according to our optimizing attempts on the double chains of compound A with PM3 method, the optimum conformation of dimer 3a wasn’t obtained, the conformation of dimmer 2a was only achieved. The reason for this phenomenon might be attributed to the existence of the α-amino groups in the carbochain, which make the chain bend toward different directions and lead to the spacial unsuitability for the carbonyl group in one chain combining with H-N group in the other chain. Moreover, based on the optimization result at ONIOM (B3LYP/6-31G:PM3) level, not only the hydrogen bond can be formed between the C=O and H-N bonds in amide group, but also can it be formed between the carbonyl and the α-amino groups, and the heptatomic ring containing two hydrogen bonds are obtained (Figure 4).

**Figure 5.**
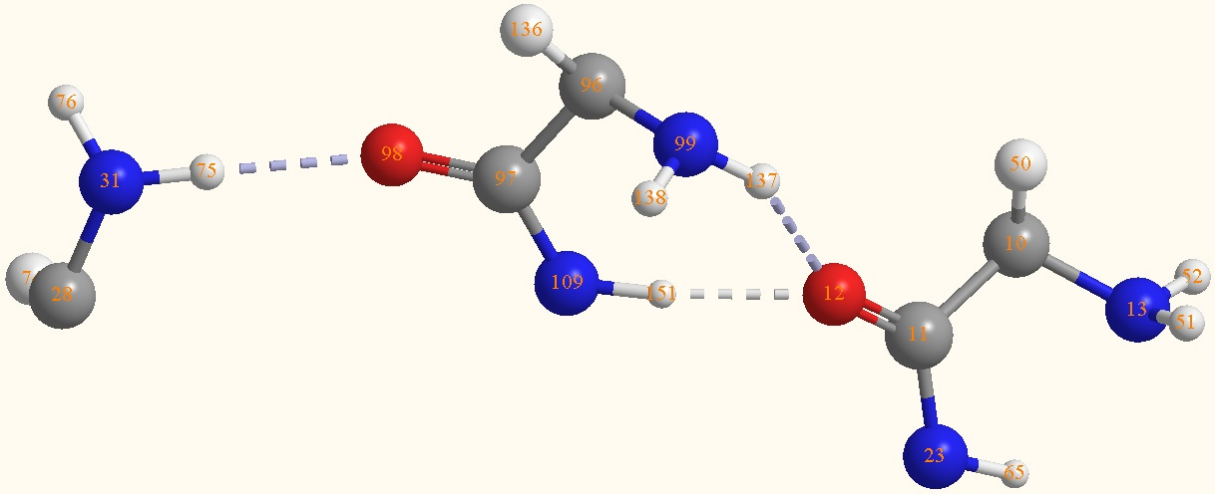
Structure of the cyclohepta bifurcated hydrogen bonds.

An interesting thing in this study was the spacial structure of the ɛ-PLL double chains. The two chains combine in a parallel β-sheet form with the hydrogen donor and acceptor groups being arranged between the two chains alternately, and the chains fold freely to match the formation of the cyclohepta bifurcated hydrogen bonds (Figure 5). This spacial structure is not only different from the double helix structure of DNA, but also not the same as the β-sheet form of the normal α-peptide chains. One chain swings around along another chain.

### 2.2. Structure of the cyclohepta bifurcated hydrogen bonds

In order to make a further comprehension of this kind of hydrogen bond, the structure of the heptatomic ring in the intermediate part of ɛ-PLL double chain is shown in Figure 5 and the main bond lengths and angles are listed in Table 1. It shows that a structure of cyclohepta-bifurcated hydrogen bonds are formed between the atoms of H(137), N(99), C(96), C(97), N(109), and H(151) from one chain and O(12) from the other chain. The two hydrogen bonds are bifurcated since they share the same oxygen atom as proton acceptor. The angle of the two hydrogen bonds is 66.6°. The hydrogen bond lengths of O (12)-H (151) and O (12)-H (137) were 1.8Å and 2.2Å, respectively. The angles of O (12)-H (151)-N (109) and O (12)-H (137)-N (99) are 166.7° and 147°, respectively, which are all in the range of 130° to 180°. The values of bond lengths and the angles suggest that these H-bonds belonged to the medium strength hydrogen bonds [38]. However, the bond length of O(12)-H(151) is much shorter than that of O(12)-H(137). The angle of O(12)-H(151)-N(109) is closer to 180° than that of O(12)-H(137)-N(99). It can be concluded that the hydrogen bond of O(12)-H(151)-N(109) is much stronger than that of O(12)-H(137). Because of the formation of the hydrogen bond, the H-N covalent bond length will be changed. The computational results show that the bond length of H(151)-N(109) (1.0276 Å) becomes longer than the normal H-N in amino group (1.022 Å), however, it is weird that the bond length of H (137)-N(99) (1.0132 Å) becomes shorter than that of the normal H-N(1.035 Å).

It also shows that one hydrogen bond is formed between the O=C of C(97)-O(98) in the cyclohepta ring and H(75)-N(31) in the other chain two residues ahead from that O(12) is located at. The distance of H(75)-O(98) is 2.02 Å and the angle of N(31)-H(75)-O(98) is 173.5°. This extra hydrogen bond is stronger than that of O(12)-H(137)-N(99). It intensifies the interaction of the two chains and is vital to the tertiary structure of the double chains.

### 2.3. Frequency analysis and IR spectra

Vibrational spectra can be extremely useful tools for the study of peptide structures and conformations [38–42]. There have also been some computational investigations on the peptide conformations based on vibration spectroscopy [36,37,43]. In this study, the frequency analysis was conducted at a higher level of ONIOM [B3LYP/6-31G (d):PM3] and the groups composing the cyclohepta bifurcated hydrogen bonds are all at the B3LYP/6-31G (d) level. Table 2 lists the main vibration frequencies of the groups shown in Figure 5. In addition, a comparison with FT-IR spectra of ɛ-PLL from experiment of Maeda *et al.* [19] was also made. Because of the formation of the hydrogen bond, the −NH_2_ asymmetrical and symmetrical stretching frequencies of N(31)-H shift toward the low frequency compared with the υ_as_ and υ_s_ of N(13)-H, however, the υ_as_ and υ_s_ of N(99)-H shift to the high frequency. The blue shift phenomenon can be attributed to the formation of bifurcated hydrogen bonds of N(99)-H(137)-O(12) and N(109)-H(151)-O(12) [44,45]. As a whole, these absorbances are very weak in intensity apart from υ_s_N(31)-H (3458 cm^−1^). The stretching vibration frequencies of N(23)-H and N(109)-H appear at 3,446 and 3,356 cm^−1^, respectively. Because there are so many absorbances in this region, a wide band forms in the range of 3,200 cm^−1^ to 3,600 cm^−1^, which agrees with the experimental data. According to Maeda *et al.*, there is a wide band with the highest absorbance at 3,382 cm^−1^, which they improperly attributed to the asymmetric stretching of −NH_2_. The absorbances of υC(11)-O(12) and υC(97)-O(98) are at 1,639 and 1,629 cm^−1^, respectively, which correlates well with the experimental result of 1,633 cm^−1^; while the rocking vibration of N (109)-H and N(23)-H are at 1,567 cm^−1^ and 1,563 cm^−1^, respectively, and are consistent with the experimental value of 1,534 cm^−1^ from Maeda *et al.* [19].

## 3. Experimental Section

In the computation processing, the molecules were first created by Chem3D software [47], then the structure optimization was sequentially conducted with MM2 and PM3 methods. On this basis, the hybrid two-level ONIOM method at B3LYP/6-31G: PM3 level was used for further optimization of the ɛ-PLL conformations. The vibrational frequencies were also calculated using a higher ONIOM [B3LYP/6-31G (d):PM3] level in order to ascertain the obtained structures or the interactions among ɛ-PLL molecules. The computational models of ɛ-PLL are supposed to be divided into two parts:

1. The polar functional groups such as the amide, amino and carbonyl groups are at the high quantum chemical level of RB3LYP/6-31G for the structure optimization.
2. All other atoms or groups such as methylene are at a semi-empirical PM3 level.

After the structure optimization, a higher quantum chemical level of ONIOM (B3LYP/6-31G(d):PM3) was used for the frequency analysis. All the calculations were performed with the Gaussian 03 series of program [48]. The optimized structures were visualized by GaussView and Chem3D, respectively.

## 4. Conclusions

Two oligomers each containing three l-lysine residues were used as model molecules for conformational simulation of the interaction of ɛ-PLL chains. Their C terminals are capped with ethylamine and N terminals are capped with α-l-aminobutanoic acid. The conformation was optimized with two level ONIOM (B3LYP/6-31G:PM3) method and the parallel β-sheet form was obtained. The two chains swing around with cyclohepta-bifurcated hydrogen bonds, which are composed of an amide and an N-H from one chain as proton donors and an oxygen of C=O from the other chain as proton acceptor. The two chains are taken as proton donors and proton acceptors alternately. The frequency analysis with the ONIOM [B3LYP/6-31G(d):PM3] shows the IR absorbances of main groups such as the amide and amidocyanogen groups are consistent with the experimental data [9].

However, there are four more points that should be made clear: (1) although the DFT method is a comparatively cheap and fast method and is extensively used to mimic the structure, inter- and intramolecular interactions of peptides or proteins, it isn’t an exact and precise method for geometry optimization, and the conformation of ɛ-PLLs needs to be confirmed by other techniques [49,50]; (2) the environment of the model molecules is different from the sample used in the FT-IR spectrum. The former is in gas phase and the latter is in solid state. It is more persuasive for evaluating the computational results when the IR spectra of the model molecules in gas phase can be obtained and used in a near future [51,52]; (3) the ɛ-polylysine is a homopolymer of ɛ-l-lysines. There are no specific corresponding locations between two chains. One section of a chain can combine with any part of the other chains around it as long as they form cyclohepta-bifurcated hydrogen bonds; (4) the oxygen proton acceptors of cyclohepta-bifurcated hydrogen bonds may come from other protein peptides. The antibacterial activity of ɛ-polylysine is generally considered to be attributed to the interactions between its positive charges and unlike charge interaction with microorganisms [53], but the interaction with cyclohepta bifurcated hydrogen bonds among the ɛ-polylysine and the microorganism proteins may give a new comprehension.

## Figures and Tables

**Table 1.** Main bond lengths and angles of the groups shown in Figure 5 obtained from ONIOM (B3LYP/6-31G: PM3).

**Atoms**	**Bond lengths/Å**	**Atoms**	**Bond angles/°**
C(11)-C(10)	1.5452	O(12)-C(11)-N(23)	125.9887
O(12)-C(11)	1.2603	C(10)-C(11)-N(23)	114.1929
N(13)-C(10)	1.4818	C(10)-C(11)-O(12)	119.8179
N(23)-C(11)	1.3531	H(51)-N(13)-H(52)	112.2561
N(31)-C(28)	1.4835	C(10)-N(13)-H(52)	115.5022
H(51)-N(13)	1.0136	C(10)-N(13)-H(51)	114.2261
H(52)-N(13)	1.0133	H(75)-N(31)-H(76)	111.7182
H(75)-N(31)	1.0221	C(28)-N(31)-H(76)	113.0713
H(76)-N(31)	1.0147	C(28)-N(31)-H(75)	114.5283
C(97)-C(96)	1.5605	C(97)-C(96)-N(99)	115.5131
O(98)-C(97)	1.2585	O(98)-C(97)-N(109)	125.1159
N(99)-C(96)	1.4676	C(96)-C(97)-N(109)	114.5476
N(109)-C(97)	1.3652	C(96)-C(97)-O(98)	120.3339
H(137)-N(99)	1.0132	H(137)-N(99)-	114.2089
H(138)-N(99)	1.0105	C(96)-N(99)-H(138)	115.0922
H(151)-N(109)	1.0276	C(96)-N(99)-H(137)	115.9221
O(12)-H(151)	1.8412	C(97)-N(109)-H(151)	119.9472
O(12)-H(137)	2.1713	H(151)-O(12)-	66.6
		O(12)-H(151)-	166.7
		O(12)-H(137)-N(99)	147
		N(31)-H(75)-O(98)	173.5

**Table 2.** Main IR frequencies of the groups shown in Figure 5 obtained from ONIOM [B3LYP/6-31G(d):PM3].

**−NH_2_ groups**	**Frequencies/cm^−^**	**Amide N-H**	**Frequencies/cm^−1^**	**C=O groups**	**Frequencies/cm^−1^**
υ_as_N(99)-H	3670vw	υN(23)-H	3446w	υC(11)-O(12)	1639w
υ_as_N(13)-H	3646 vw	υN(109)-H	3356m	υC(97)-O(98)	1629m
υ_as_N(31)-H	3592 vw	ρN(109)-H	1567s		
υ_s_N(99)-H	3551 vw	ρN(23)-H	1563w		
υ_s_N(13)-H	3538 w				
υ_s_N(31)-H	3458m				
δN(31)-H	1711vw				
δN(13)-H	1686vw				
δN(99)-H	1679vw				
δC(28)-H	1316vw				
δC(96)-H	1315vw				
δC(10)-H	1328vw				

υ: Stretching vibration; δ: Bending vibration; ρ: Rocking vibration; v: Very; w: Weak; m: Middle; s: Strong.
